# Dual-surface aberration-increasing lenses versus single-vision lenses in non-myopic children: a randomized clinical trial

**DOI:** 10.1186/s40662-026-00486-0

**Published:** 2026-04-27

**Authors:** Weiwei Lu, Shiqi Zhou, Lihua Yu, Renai Chen, Ruru Chen, Yang Zhao, Lan Wang, Yi Chen, Fan Lu, Jiawei Zhou, Wanqing Jin

**Affiliations:** 1 School of Ophthalmology and Optometry and Eye Hospital, and State Key Laboratory of Ophthalmology, Optometry and Vision Science, Wenzhou Medical University, 270 Xueyuan Road, Wenzhou, 325027 Zhejiang People’s Republic of China; 2 National Clinical Research Center for Ocular Diseases, Eye Hospital, Wenzhou Medical University, Wenzhou, China

**Keywords:** Aberration, Myopia, Emmetropia, Mild hyperopia, Spectacles

## Abstract

**Purpose:**

To investigate the 1-year preventive efficacy of dual-surface aberration-increasing (DSAI) lenses on axial elongation and refractive change in non-myopic children.

**Methods:**

This was a 2-year randomized, controlled, prospective trial with a planned interim analysis at the 1-year follow-up to assess the preliminary efficacy and safety of DSAI lenses. One hundred children aged 6–12 years with cycloplegic spherical equivalent refraction between −0.50 diopters (D) and + 2.00 D, classified as non-myopic, were recruited. Participants were randomly assigned to wear either DSAI spectacle lenses or single-vision (SV) spectacle lenses in daily life. The primary outcomes were axial elongation and refractive change relative to baseline measurements, with axial elongation considered the main structural outcome in the results.

**Results:**

Enrolment commenced in June 2023. One hundred participants were recruited, and 92 (DSAI group: n = 46, mean ± standard deviation age: 8.15 ± 1.49 years; SV group: n = 46, 8.32 ± 1.62 years) completed the 1-year follow-up. No intervention-related adverse events were observed during the study period. Participants in the DSAI group exhibited significantly less axial elongation (difference: 0.15 mm; 95% confidence interval [CI]: 0.06 to 0.24 mm; *P* = 0.002) and refractive change (difference: −0.26 D; 95% CI: −0.50 to −0.02 D; *P* = 0.032) than those in the SV group. Subgroup analysis showed that emmetropic children, older children (8.1–12.0 years), and those wearing lenses for longer durations (≥ 11 h/day) in the DSAI group had significantly less model-adjusted axial elongation than the corresponding SV subgroups (all *P* < 0.05).

**Conclusion:**

The 1-year interim results suggest that DSAI lenses help to slow axial elongation and refractive change in non-myopic children, with a relatively greater effect observed in emmetropic children, older children, and those who wore the lenses for longer durations.

**Trial Registration:**

This trial is registered at Chinese Clinical Trial Registry with trial registration number: ChiCTR2300078464.

**Supplementary Information:**

The online version contains supplementary material available at 10.1186/s40662-026-00486-0.

## Background

The prevalence of myopia is rising rapidly worldwide [1], with an increasing incidence at younger ages, particularly in Asia [2, 3]. By 2021, myopia affected 51.9% of first- to third-grade primary school students in southern China [4]. Early-onset myopia is more likely to progress to high myopia [3, 5], thereby increasing the risk of ocular diseases such as retinal atrophy [6], retinal detachment [7], and macular retinoschisis [8]. Therefore, early prevention of myopia in childhood to delay its onset is essential to reduce long-term refractive error and mitigate its impact on individuals and society.

Current research on myopia prevention remains limited. Increasing outdoor activity is a well-recognized strategy for delaying its onset [9–11]; however, extreme weather conditions, such as excessive heat or cold, may restrict children’s participation. Atropine eye drops have also attracted research interest; however, their optimal dosage and effectiveness remain uncertain [12, 13]. Additionally, atropine eye drops may cause adverse effects on accommodation and pupil size [14–17]. Given these uncertainties, exploring safer and more convenient strategies for myopia prevention is essential.

Specially designed spectacle lenses have been widely studied for their effectiveness in slowing myopia progression in children [18–21]. Lenses with contiguous high aspherical lenslets (HALs) generate peripheral myopic defocus signals, which effectively slow axial elongation in myopic children [20]. Other designs, such as lenses with cylindrical annular refractive elements, increase total ocular aberrations to control refractive progression [19]. However, most of these lenses feature a single-surface design with one myopia control mechanism and are primarily intended for children already diagnosed with myopia. A study investigated the effects of lenses featuring HALs on the front surface in slowing axial elongation and refractive change in children who were not yet myopic [22]. However, no significant effect was detected, which may have been due to insufficient lens-wearing time [22]. This finding suggests that more intensive interventions may be required for effective myopia prevention.

In this study, dual-surface aberration-increasing (DSAI) lenses that induce both myopic defocus signals and multiple types of aberrations were developed. An exploratory, randomized controlled trial was designed to compare the effectiveness of DSAI lenses with single-vision (SV) lenses for myopia prevention. Axial elongation was assessed as the primary structural outcome, and refractive change was also reported as a co-primary outcome.

## Methods

### Study design

This 2-year prospective, randomized controlled trial, initiated in June 2023, was conducted at the Eye Hospital of Wenzhou Medical University, China, to investigate the potential role of DSAI lenses in early myopia management. Eligible participants were randomly assigned (1:1) to wear either DSAI lenses (intervention group) or standard SV lenses (control group) according to a computer-generated allocation sequence. Simple randomization was performed by a clinical research coordinator who was not involved in recruitment, clinical examinations, data collection, or analysis. All lenses were identically packaged and distributed by the coordinator to lens dispensers. Except for the coordinator, all other investigators (including examiners, dispensers, and statisticians), as well as participants and their guardians, remained masked throughout the study. For safety monitoring, after the 1-year follow-up, de-identified data were provided to statisticians for a planned interim analysis to assess the preliminary effects of DSAI lenses, while masking was maintained for all other investigators, participants, and guardians.

Follow-up visits were scheduled at baseline, 1 month, 3 months, and every 3 months thereafter. Written informed consent was obtained from participants and their guardians after an explanation of the study’s purpose, procedures, potential risks, and benefits. Detailed research methodology and the study protocol are provided in Additional file 1. The study was approved by the Ethics Committee of the Eye Hospital of Wenzhou Medical University (2022-211-K-166-01) and adhered to the tenets of the Declaration of Helsinki. This trial is registered at Chinese Clinical Trial Registry with trial registration number ChiCTR2300078464.

### Participants

Potentially eligible participants were recruited from hospital outpatient clinics. The inclusion criteria were as follows: (1) age 6–12 years; (2) spherical equivalent refraction (SER) between −0.50 D and + 2.00 D, measured using an autorefractor under cycloplegia; (3) astigmatism ≤ 1.50 D; (4) distance best-corrected visual acuity (BCVA) of 0.1 logMAR or better in each eye; (5) anisometropia ≤ 1.50 D; and (6) willingness to wear spectacle lenses. The exclusion criteria were as follows: (1) intraocular pressure ≥ 21 mmHg; (2) manifest strabismus; (3) ocular or systemic diseases affecting visual development (excluding refractive errors); (4) a history of ocular or systemic surgery affecting visual development; (5) use of defocus spectacle lenses or low-concentration atropine eye drops within the previous 3 months; (6) use of orthokeratology lenses or other products (e.g., red light-related treatment methods) intended to prevent or control myopia within the previous 6 months; (7) a history of systemic medications affecting growth and development (e.g., growth hormone); (8) concurrent participation in clinical trials related to myopia control or prevention; and (9) other conditions deemed unsuitable for the trial by the investigator.

### Sample size

The sample size was calculated using SPSS (IBM Inc., Armonk, NY, United States) based on a two-sample t-test assuming equal variances. The calculation was based on axial elongation, which is closely associated with myopia progression and related retinal complications [23, 24]. The mean ± standard deviation (SD) annual axial elongation was assumed to be 0.36 ± 0.23 mm in the control group, based on previous studies of non-myopic children aged 6–12 years [12, 25–27]. The intervention effect of the DSAI lenses (as defined in the statistical analysis section) was assumed to be 40%. This anticipated effect size was estimated from the control rate of axial elongation reported in a study using spectacle lenses incorporating diffusion optics technology [28]. Assuming a 10% drop-out rate, a minimum sample size of 94 participants (47 per group) was required to achieve 80% power at a significance level of 0.05.

### Interventions

Both the anterior and posterior surfaces of the DSAI lenses feature a central optical zone (10.13 mm) for refractive error correction, thereby ensuring clear central vision. The peripheral zone integrates two design elements. The anterior surface incorporates a concentric microlens array that generates myopic defocus signals anterior to the peripheral retina. Microlenses with different refractive powers are arranged in 11 concentric rings (+ 5.00 D in the three inner rings, + 5.50 D in the four intermediate rings, and + 6.00 D in the four outer rings; see Figure S1 in Additional file 2 for details). The posterior surface features a radial refractive gradient that induces a range of lower- and higher-order aberrations (see Figure S1 in Additional file 2 for the corresponding Zernike polynomials and representative aberrations), thereby further increasing total ocular aberrations beyond those introduced by the anterior surface. The sagittal height map of the posterior surface is shown in Figure S2 (Additional file 2). The design is protected under the Patent Cooperation Treaty (202122046858X and 202221884655.6). Compared with single-surface lenses incorporating microlens arrays, the modulation transfer function (MTF) of the DSAI lenses decreases more markedly as spatial frequency increases (Figure S3 in Additional file 2).

Frames with larger, more rounded lens rims were selected to ensure full coverage of the optical elements of the DSAI lenses. Each participant’s pupillary distance and fitting height were measured precisely, and trained opticians assisted in frame selection to ensure optimal lens centration. All frames were fitted with adjustable nose pads and ear hooks to prevent slippage. Participants were encouraged to wear the spectacles full-time in daily life. The relative position of the pupil and the frame was assessed at baseline and at each follow-up visit; any misalignment was corrected promptly to maintain optimal lens positioning throughout the study period.

### Trial outcomes

The primary outcome was axial elongation from baseline, reflecting the structural changes underlying myopic drift. Refractive change was defined as a co-primary outcome to provide complementary information. Secondary outcomes included the incidence of myopia and average daily lens-wearing time. Axial length (AL) was measured before cycloplegia using the IOLMaster 700 (Zeiss, Germany). Cycloplegia was induced with 0.5% proxymetacaine hydrochloride (one drop) and 1% cyclopentolate (three drops) administered at 5-min intervals. The status of cycloplegia was evaluated at least 30 min after the final drop of cyclopentolate. Criteria for complete cycloplegia included a pupil diameter > 6 mm, absence of a pupillary light response, and lack of accommodative response assessed by dynamic retinoscopy. Once cycloplegia was confirmed, refractive error was measured using an automated refractor (Topcon KR-800, Topcon Corporation, Japan) and converted to SER. An objective SER of −0.50 D or more myopic under cycloplegia was defined as myopia [29]. Cycloplegic SER was measured at lens dispensing (baseline) and at the 6- and 12-month follow-up visits, whereas AL was measured at baseline and at the 1-, 3-, 6-, 9-, and 12-month follow-up visits.

Self-assessment questionnaires (Table S1 in Additional file 2) were designed to record lens-wearing duration (items 1–2), daily schedules (items 3–8), and subjective adaptation to the lenses (items 9–10). The average daily hours spent wearing lenses, sleeping, using electronic devices, and engaging in outdoor activities were calculated using the formulae provided in Additional file 2. Subjective lens adaptation was assessed at dispensing and at the 1- and 3-month follow-up visits, whereas the other items were reported at each follow-up visit. Furthermore, distance BCVA and contrast sensitivity (CS) were assessed as indicators of objective adaptation to the lenses. Distance BCVA (5 m) was measured monocularly at baseline and every 3 months thereafter using a standard logarithmic visual acuity chart under bright, uniform lighting conditions. CS at spatial frequencies of 3, 6, 12, and 18 cycles per degree was measured at baseline and at the 6- and 12-month follow-up visits. Testing was conducted using the CSV-1000 tester (VectorVision, Ohio, USA) at a viewing distance of 2.5 m under both mesopic (3 cd/m^2^) and photopic (85 cd/m^2^) conditions.

### Statistical analysis

Statistical analyses were performed using SPSS (IBM Inc., Armonk, NY, United States). Normality was assessed using the Kolmogorov–Smirnov test. Normally distributed data are presented as the mean ± SD or mean ± standard error. Intergroup differences in continuous variables were analysed using independent-samples t-tests. Categorical variables are presented as percentages and were analysed using Chi-square tests. Primary outcomes, including axial elongation and refractive change, were calculated as the differences between each follow-up measurement and baseline. The analysis focused on data obtained at the 6- and 12-month follow-up visits. The suppression effect size of the DSAI lenses on axial elongation or refractive change was calculated according to the following formula:

$$Effect\,size = \frac{Changes\,in\,the\,SV\,group - Changes\,in\,the\,DSAI\,group}{{Changes\,in\,the\,SV\,group}} \times 100$$

Due to the high interocular correlation (axial elongation: R = 0.79, *P* < 0.001; refractive change: R = 0.60, *P* < 0.001), only right-eye data were analysed, following the approach adopted in previously published clinical trials [18, 20]. A univariate general linear model was applied to estimate axial elongation and refractive change after adjustment for gender, age, lens-wearing time, initial AL (or initial cycloplegic SER), photopic pupil size, daily duration of outdoor activities and electronic device use, as well as the number of myopic parents. To estimate adjusted myopia incidence, a logistic regression model was employed, controlling for potential confounders including gender, age, lens-wearing time, initial cycloplegic SER, photopic pupil size, daily duration of outdoor activities, time spent using electronic devices, and the number of myopic parents. Diagnostic checks, including model fit evaluation and multicollinearity assessment, confirmed that all model assumptions were satisfied. Both adjusted and unadjusted analyses were performed to ensure the robustness of the assessment of intergroup differences. Subgroup analyses were conducted for adjusted axial elongation and refractive change according to initial refractive status (emmetropia, −0.50 D < cycloplegic SER < 0.50 D; mild hyperopia, 0.50 D ≤ cycloplegic SER ≤ 2.00 D) [29, 30], age (6.0–8.0 and 8.1–12.0 years), and daily lens-wearing time (< 11 and ≥ 11 h/day). As this was an exploratory study, *P* < 0.05 was considered to indicate statistical significance [20, 22]. To control for type I error arising from multiple comparisons, *P* < 0.025 was considered statistically significant when analysing 6-month follow-up and subgroup data.

## Results

**Fig. 1:**
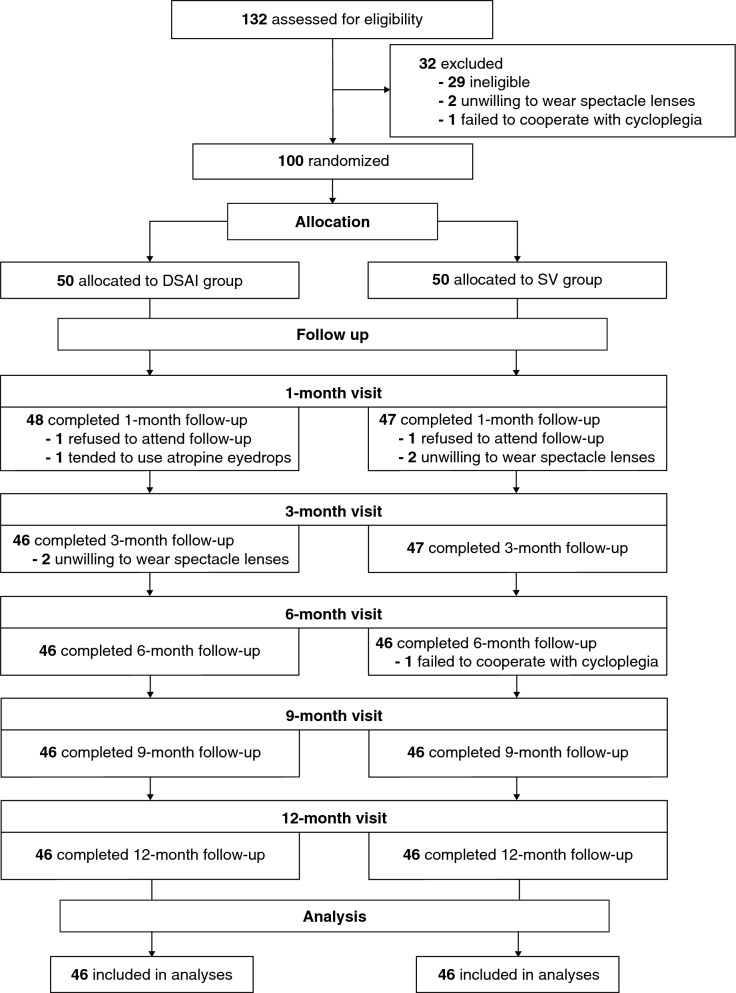
Study design and participant flow at each visit. DSAI, dual-surface aberration-increasing; SV, single-vision

A total of 100 children were recruited and randomly assigned to the DSAI (n = 50) or the SV group (n = 50). None of the participants had undergone previous interventions for myopia prevention. Figure 1 presents the flow chart of the participants. Ninety-two participants were included in this interim analysis after 1 year of follow-up (46 in each group). Eight children withdrew for reasons unrelated to the DSAI lens design, including unwillingness to wear spectacles (n = 4), refusal to attend follow-up visits (n = 2), reluctance to undergo cycloplegia (n = 1), and opting for alternative myopia prevention strategies (n = 1). Baseline characteristics (Table 1), daily routines (including time spent on outdoor activities, electronic device use, and sleep), and lens-wearing time over 12 months (Table S2 in Additional file 2) were analysed, and no significant differences were observed between the groups (all *P* > 0.05). The distributions of initial cycloplegic SER in the two groups are shown in Figure S4 (*P* = 0.44; Additional file 2). The average daily lens-wearing time was 11.09 ± 1.07 h in the DSAI group and 10.97 ± 1.46 h in the SV group (*P* = 0.67).

**Table 1:** Baseline characteristics of participants who completed the 12-month follow-up

Characteristics	DSAI group (n = 46)	SV group (n = 46)	df	t/χ^2^ value	*P* value
Age (years)	8.15 [1.49]	8.32 [1.62]	90	0.51	0.61
Gender					
Boys, number (%)	20 (43.5)	22 (47.8)	1	0.04	0.83
Girls, number (%)	26 (56.5)	24 (52.2)			
Cycloplegic SER (D)	0.29 [0.58]	0.38 [0.57]	90	0.77	0.44
AL (mm)	23.17 [0.68]	23.17 [0.79]	90	−0.05	0.96
Mean K (D)	43.29 [1.34]	43.49 [1.28]	90	0.76	0.45
AL/CR	2.97 [0.05]	2.98 [0.06]	90	1.13	0.26
IOP (mmHg)	15.44 [2.20]	15.79 [2.52]	90	0.69	0.49
Photopic pupil size (mm)	4.36 [0.61]	4.13 [0.87]	90	−1.43	0.16
CCT (µm)	546.93 [33.62]	538.36 [32.17]	90	−1.24	0.22
Parents with high myopia					
0, number (%)	7 (15.2)	3 (6.5)	2	1.80	0.41
1, number (%)	14 (30.4)	15 (32.6)			
2, number (%)	25 (54.3)	28 (60.9)			

Data are presented as mean [SD] or number (%)

*DSAI* = dual-surface aberration-increasing; *SV* = single-vision; *df* = degrees of freedom; *SER* = spherical equivalent refraction; *D* = diopter; *AL* = axial length; *K* = keratometry; *CR* = corneal radius; *IOP* = intraocular pressure; *CCT* = central corneal thickness

### Overall comparison of axial elongation and refractive change between the DSAI and SV groups

**Fig. 2:**
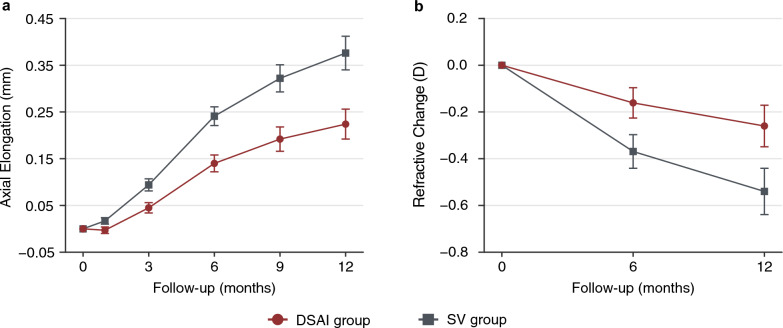
Model-adjusted axial elongation (**a**) and refractive change (**b**) in the two groups from baseline to the 12-month follow-up. The adjustment variables included in the univariate general linear model were gender, age, lens-wearing time, initial axial length (or cycloplegic spherical equivalent refraction), photopic pupil size, number of parents with myopia, and daily durations of outdoor activities and electronic device use. Significant differences in both axial elongation and refractive change were observed between the DSAI and SV groups at each time point. Error bars represent the standard error. DSAI, dual-surface aberration-increasing; SV, single-vision; D, diopter

The unadjusted mean axial elongation at the 6- and 12-month follow-up visits is presented in Table S3 (Additional file 2) and Table 2, respectively. After 1 year of intervention, the DSAI group showed significantly less axial elongation than the SV group (difference: 0.15 ± 0.05 mm; 95% confidence interval [CI]: 0.06 to 0.24 mm;* P* = 0.002). The suppression rate of the DSAI lenses for axial elongation was 38.5%. In the univariate general linear model analysis, group (*P* < 0.001), pupil size (*P* = 0.002), age (*P* = 0.006), and gender (*P* = 0.004) were significantly associated with axial elongation. Figure 2a presents the model-adjusted axial elongation over 1 year. A significant intergroup difference remained at the 12-month follow-up after adjustment for confounders (DSAI group: 0.22 ± 0.03 mm; SV group: 0.38 ± 0.04 mm; *P* < 0.001). The adjusted suppression rate was 42.1%.

**Table 2:** Unadjusted axial elongation and refractive change in each group at the 12-month follow-up

Outcome measures	DSAI group (n = 46)	SV group (n = 46)	Mean difference	95% CI	df	t value	*P* value
Axial elongation (mm)	0.24 ± 0.03	0.39 ± 0.04	0.15 ± 0.05	0.06 to 0.24	90	3.27	**0.002**
Refractive change (D)	−0.32 ± 0.06	−0.59 ± 0.10	−0.26 ± 0.12	−0.50 to −0.02	75	−2.19	**0.032**

*DSAI* = dual-surface aberration-increasing; *SV* = single-vision; *CI* = confidence interval; *df* = degrees of freedom; *D* = diopter. Data are presented as mean ± SE

*P* values in bold indicate statistical significance

Unadjusted mean refractive changes at the 12-month follow-up are also presented in Table 2. After 1 year of intervention, the DSAI group exhibited a significantly smaller myopic shift than the SV group (difference: −0.26 ± 0.12 D; 95% CI: −0.50 to −0.02 D; *P* = 0.032; suppression rate: 45.8%). In the univariate general linear model, group (*P* = 0.022) and pupil size (*P* = 0.007) were significantly associated with refractive change. The model-adjusted refractive change in the DSAI group was significantly less than that in the SV group (−0.26 ± 0.09 D vs. −0.54 ± 0.10 D; *P* = 0.022; Fig. 2b), with a suppression rate of 51.9%.

After 1 year of intervention, the intergroup differences in the distributions of axial elongation and refractive change were statistically significant (axial elongation: X^2^ = 9.75, *P* = 0.02; refractive change: X^2^ = 11.07, *P* = 0.02) (Figure S5 in Additional file 2). Rapid axial elongation (> 0.50 mm from baseline) was less common in the DSAI group than in the SV group (DSAI group: 6%; SV group: 24%; difference: 18% ± 6%; *P* < 0.05). Furthermore, 11% (5/46) of children in the DSAI group exhibited no axial elongation (≤ 0 mm), which was significantly higher than that in the SV group (difference: 11% ± 5%; *P* < 0.05). Similarly, rapid myopic drift (> 1.0 D from baseline) occurred in 2% (1/46) of participants in the DSAI group compared with 24% (11/46) in the SV group (difference: 22% ± 2%; *P* < 0.05).

### Subgroup analyses based on initial refractive status

Table 3 presents the results of the subgroup analysis of adjusted axial elongation and refractive change at the 12-month follow-up, stratified by initial refractive status. Among emmetropic children, participants in the DSAI group exhibited significantly less axial elongation (difference: 0.23 ± 0.05 mm; 95% CI: 0.12 to 0.34 mm; *P* < 0.001) and refractive change (difference: −0.43 ± 0.16 D; 95% CI: −0.75 to −0.11 D; *P* = 0.010) than those in the SV group. Confounders associated with axial elongation and refractive change in the univariate general linear model analysis are detailed in Additional file 2. The incidence of myopia was 44.8% in the DSAI group and 65.4% in the SV group (difference: 20.6%; 95% CI: −5.1% to 46.3%; *P* = 0.18). In the logistic regression analysis, the model-adjusted risk of myopia was significantly lower in the DSAI group than in the SV group (odds ratio [OR] = 0.14; 95% CI: 0.03 to 0.67; *P* = 0.015).

**Table 3:** Results at the 12-month follow-up of subgroup analysis based on initial refractive status

Outcome measures	DSAI group	SV group	Mean difference	95% CI	df	F value	*P* value
Axial elongation (mm)							
Emmetropia	0.23 ± 0.04 (n = 29)	0.46 ± 0.04 (n = 26)	0.23 ± 0.05	0.12 to 0.34	1	18.57	** < 0.001**
Mild hyperopia	0.21 ± 0.05 (n = 17)	0.27 ± 0.05 (n =20)	0.06 ± 0.05	−0.05 to 0.16	1	1.25	0.27
Refractive change (D)							
Emmetropia	−0.30 ± 0.11 (n = 29)	−0.73 ± 0.13 (n = 26)	−0.43 ± 0.16	−0.75 to −0.11	1	7.27	**0.010**
Mild hyperopia	−0.22 ± 0.16 (n = 17)	−0.28 ± 0.17 (n = 20)	−0.06 ± 0.18	−0.43 to 0.31	1	0.10	0.75

*DSAI* = dual-surface aberration-increasing; *SV* = single-vision; *CI* = confidence interval; *df* = degrees of freedom; *D* = diopter. Data are presented as mean ± SE, adjusted for confounders including gender, age, lens-wearing time, initial axial length (or cycloplegic spherical equivalent refraction [SER]), photopic pupil size, number of parents with myopia, and daily durations of outdoor activities and electronic device use. Emmetropia: −0.50 D < initial cycloplegic SER < 0.50 D; mild hyperopia: 0.50 D ≤ initial cycloplegic SER ≤ 2.00 D

*P* values in bold indicate statistical significance

Among children with initial mild hyperopia, no significant differences were observed in axial elongation (difference: 0.06 ± 0.05 mm; 95% CI: −0.05 to 0.16 mm; *P* = 0.27) or refractive change (difference: −0.06 ± 0.18 D; 95% CI: −0.43 to 0.31 D; *P* = 0.75) between the DSAI and SV groups. The incidence of myopia was 0.0% in the DSAI group and 10.0% in the SV group (difference: 10.0%; 95% CI: −9.8% to 30.1%; *P* = 0.49). Logistic regression analysis also showed no significant intergroup difference in adjusted myopia risk (*P* = 0.99).

### Subgroup analyses based on age and lens-wearing time

Table S4 in Additional file 2 presents the results of the subgroup analyses stratified by age and daily lens-wearing time. Older children (8.1–12.0 years) in the DSAI group showed significantly less axial elongation (difference: 0.19 ± 0.05 mm; 95% CI: 0.09 to 0.30 mm; *P* < 0.001) and refractive change (difference: −0.38 ± 0.14 D; 95% CI: −0.65 to −0.10 D; *P* = 0.009) than those in the SV group. Among participants with longer lens-wearing durations (≥ 11 h/day), the DSAI group exhibited significantly less axial elongation than the SV group (difference: 0.15 ± 0.06 mm; 95% CI: 0.04 to 0.26 mm; *P* = 0.009), whereas refractive change was comparable between the groups. Among younger children (6.0–8.0 years) or those with shorter lens-wearing durations (< 11 h/day), neither axial elongation nor refractive change differed significantly between the groups.

### Adverse events and adaptation

Throughout the 1-year intervention, no adverse events related to the DSAI lenses were reported. There were no significant intergroup differences in the change in corneal or total astigmatism at the 12-month follow-up (corneal astigmatism: difference −0.02 ± 0.06 D, t = −0.48, *P* = 0.63; total astigmatism: difference 0.10 ± 0.06 D, t = 1.77,* P* = 0.08). Both subjective and objective adaptation to the lenses were evaluated. At the 1-month follow-up, 96% of participants in the DSAI group reported no discomfort while wearing the lenses in the adaptation questionnaire. The comfort rate in the DSAI group did not differ significantly from that in the SV group (difference: 4%; *P* = 0.48; Table S5 in Additional file 2). Similarly, no statistically significant difference in subjective visual clarity was observed between the two groups (*P* > 0.99; Table S5 in Additional file 2). After 1 year of lens wear, distance BCVA in the DSAI group was comparable to that in the SV group (t = −1.37, *P* = 0.17). Log CS values across different spatial frequencies were fitted to generate a CS function, and the area under the log CS function was compared between the two groups. No significant intergroup differences were observed under either mesopic or photopic conditions at the 6- and 12-month follow-up visits (see Table S6 in Additional file 2 for detailed data).

## Discussion

This randomized clinical trial evaluated the effectiveness of dual-surface lenses (DSAI lenses) in slowing axial elongation and refractive change in non-myopic children. The present paper reports the interim results of the trial. After 1 year of intervention, children in the DSAI group exhibited less axial elongation and a smaller myopic shift in refraction than those in the SV group. The effect was particularly notable in emmetropic children, older children (8.1–12.0 years), and those who wore the lenses for more than 11 h/day.

In contrast to previously reported myopia control lenses, which have mostly focused on single-surface modifications [20, 21, 31], DSAI lenses employ a dual-surface strategy. The concentric microlens array on the anterior surface provides myopic defocus signals to the peripheral retina, and its efficacy in myopia control has been demonstrated in both animal models [32] and human clinical trials [20]. The radial refractive gradient on the posterior surface increases higher-order aberrations, such as spherical aberration (see Table 2 in Additional file 1). These aberrations may provide odd-error cues that help discriminate the direction of defocus signals [33]. Studies of myopic children undergoing orthokeratology have also reported negative correlations between axial elongation and changes in positive spherical aberration [34]. In addition, the design of DSAI lenses reduces MTF values compared with single-surface lenses incorporating microlens arrays, reflecting reduced image contrast on the peripheral retina. According to the Neitz theory, spurious high-contrast signalling caused by mutations in photopigment genes has been proposed as a contributor to common myopia [35]. Beyond this theory, emerging clinical evidence suggests that contrast reduction itself may serve as a signal for myopia inhibition independent of myopic defocus [18, 21]. Its potential role as a mechanism for slowing myopia progression has also been highlighted in a recent NASEM report. Taken together, these factors may help explain the observed effectiveness of DSAI lenses.

Whereas previous studies induced myopia by degrading the retinal image [36], such effects were typically achieved through full-field form deprivation using high-power diffusers [37]. However, DSAI lenses provide clear central vision and adequate light transmittance while selectively manipulating peripheral visual quality. This distinction is critical, as research has shown that daily removal of diffusers and subsequent light exposure can inhibit the development of form-deprivation myopia [38, 39]. Furthermore, regular assessment of lens centration at each follow-up visit ensured minimal impact of peripheral blur on both subjective and objective visual quality throughout the study period. In addition, the observed control effect on axial elongation and refractive change suggests that the optical design of DSAI lenses imposes a relatively small visual burden on participants.

The study provides preliminary insights into individualised myopia prevention. DSAI lenses demonstrated greater control of axial elongation and refractive change in emmetropic children than in those with mild hyperopia. However, the intergroup comparison revealed a methodological discrepancy in myopia incidence among emmetropic children that could not be attributed to baseline refractive differences between the groups (DSAI group: −0.07 ± 0.30 D; SV group: −0.02 ± 0.25 D; *P* = 0.486). Although the underlying causes remain to be clarified, this inconsistency may be reduced or resolved in the ongoing follow-up assessments. With respect to age-stratified analysis, the intervention effect of DSAI lenses was more pronounced in older children (8.1–12.0 years). To date, evidence regarding age-related responses to myopia interventions remains inconsistent [23]. Whereas some studies have reported a trend similar to that observed in the present study [40, 41], others have reported stronger responses in younger cohorts [42, 43] or no significant age dependence [23]. In the current trial, the stronger intervention effect observed among older children may be partly attributable to the higher proportion of emmetropic participants in this subgroup (older group: 76%; younger group: 42%), who appeared to derive greater benefit from DSAI lenses. This finding suggests a potential association between baseline refractive status and treatment response. A similar pattern of varying efficacy across populations with different baseline refractive ranges has been observed in studies using HAL lenses [20, 22, 44]. While HAL lenses effectively controlled axial elongation in myopic children (−4.75 to −0.75 D) [20], their efficacy was relatively modest in children who were less myopic (−0.50 to +2.00 D) [22, 44]. Although the effect of DSAI lenses reported in the present study was less pronounced than that of HAL lenses in myopic children (< −0.75 D) [20], it appears more favourable in non-myopic children (≥ 0.00 D) [22]. Therefore, for children retaining hyperopic reserves, regular monitoring of AL and refraction is necessary to guide the timely initiation of preventive strategies in clinical practice. Beyond the factors mentioned above, longer lens-wearing duration also enhanced the effect of DSAI lenses, consistent with the dose–response patterns reported in studies using other specially designed lenses for myopia control [20, 27, 45]. Given the exploratory nature of these subgroup analyses, these findings should be interpreted with caution, and further studies are required to determine the optimal timing and protocol for myopia prevention.

Several limitations of the current study should be acknowledged. First, although the average daily wearing time in the DSAI group was 11.09 h, no specific daily or weekly wearing schedule was prescribed at the time of lens dispensing. Data on lens-wearing time relied on self-report questionnaires and may therefore be subject to recall bias. Objective tools, such as electronic monitoring devices, should be incorporated in future studies to more accurately track participant compliance. Second, refractive changes were measured under cycloplegia, which may not fully reflect natural visual conditions in daily life. Previous studies have shown that ocular accommodation during near work can modulate the peripheral refraction pattern [46, 47]. Specifically, the myopic shift in the spherical component and the marked reduction in the J0 astigmatic component [48] may further alter the optical signals generated by the anterior and posterior surfaces of the DSAI lenses. In this context, future studies incorporating peripheral refraction measurements under natural viewing conditions may help clarify the interplay between accommodation and the lens-induced optical profile in myopia control. Third, the DSAI and SV lenses differ subtly in appearance; however, these differences can be discerned only under specific lighting conditions and from particular viewing angles. To minimize the risk of unmasking, neither investigators nor participants were informed how to identify the optical elements embedded in the DSAI lenses, and all examiners were instructed not to intentionally inspect lens structures at each follow-up visit. It should be acknowledged that some participants in the DSAI group may have had the opportunity to notice the presence of optical elements. The effectiveness of masking will be evaluated by calculating Bang’s index at the end of the trial [49].

The current analysis confirms the preliminary efficacy of DSAI lenses in slowing axial elongation and refractive change in non-myopic children. No severe adverse events related to DSAI lenses have been reported to date, and participants demonstrated an acceptable level of adherence to lens wear. Ongoing follow-up assessments are being conducted to evaluate the long-term benefits of DSAI lenses in myopia management. As an exploratory clinical trial designed to assess the general applicability of the lenses, the recruitment criteria were relatively inclusive compared with those of confirmatory trials. Consequently, studies targeting specific populations are warranted to further verify the subgroup findings and refine personalized intervention strategies. Although DSAI lenses integrate optical elements on both the anterior and posterior surfaces, the present study evaluated the overall effect of the combined design. It is essential to isolate the contribution of each surface and systematically compare their individual and combined effects. Regarding visual performance, both subjective and objective measures showed no significant intergroup differences, consistent with findings for perifocal lenses that also incorporate a refractive gradient element [50]. Future studies could further compare DSAI lenses with lenslet-based designs [51] or other optical concepts to investigate their effects on different aspects of visual function, such as light disturbance [50] or stereopsis [52]. Given that children wearing these specially designed lenses tend to adopt head-movement strategies to maintain clear vision through the central optical zone [53], assessment of visual behaviour during lens wear is also essential. Such investigations would facilitate optimisation of spectacle lens design for myopia management and support the development of more targeted optical interventions.

## Conclusions

The study demonstrated good compliance and adaptability to DSAI lenses among participants. Preliminary findings indicate that DSAI lenses may have a beneficial effect in controlling axial elongation and refractive change in non-myopic children. Based on the subgroup analyses, the effect appears to be more pronounced in emmetropic children than in those with mild hyperopia.

## Supplementary Information

Supplementary Material 1.

Supplementary Material 2.

## Data Availability

The datasets used and/or analysed during the current study are available from the corresponding author upon reasonable request.
