# Supplementary material for: Dual-surface aberration-increasing lenses versus single-vision lenses in non-myopic children: a randomized clinical trial
Source: Eye Vis (Lond). 2026 Apr 27;13:15. doi: 10.1186/s40662-026-00486-0 (PMC13112723; doi:10.1186/s40662-026-00486-0)
Supplement: Supplementary file 1 — Supplementary Material 1. [file 40662_2026_486_MOESM1_ESM.docx]

**Aberration-enhanced dual-surface lens versus single-vision lens in emmetropic and mildly hyperopic children: a randomized clinical trial protocol**

Contents

1. Investigators

2. Study site

3. Background

4. Hypothesis

5. Study objectives

6. Materials and methods

6.1 Study design

6.2 Scheme

6.3 Enrolment and eligibility

6.4 Sample size

6.5 Randomisation and masking

6.6 Interventions

6.7 Study outcomes

6.8 Study visit schedule

6.9 Outcome measurements

7. Data management

8. Statistical analysis

9. Ethics and governance

1. **Investigators**

**Principal investigator:**

Wanqing Jin, MMed, Chief Physician, Associate Professor, Ophthalmologist and Optometrist
Affiliation: School of Ophthalmology and Optometry and Eye Hospital, State Key Laboratory of Ophthalmology, Optometry and Vision Science, National Clinical Research Center for Ocular Diseases, Wenzhou Medical University

Address: 270 Xueyuan West Road, Lucheng District, Wenzhou, Zhejiang, China, 325027

Jiawei Zhou, PhD, Professor and Director, Zhejiang–Canada Translational Vision Research Center

Affiliation: School of Ophthalmology and Optometry and Eye Hospital, State Key Laboratory of Ophthalmology, Optometry and Vision Science, National Clinical Research Center for Ocular Diseases, Wenzhou Medical University

Address: 270 Xueyuan West Road, Lucheng District, Wenzhou, Zhejiang, China, 325027

Weiwei Lu, MMed, Ophthalmologist and Optometrist
Affiliation: School of Ophthalmology and Optometry and Eye Hospital, State Key Laboratory of Ophthalmology, Optometry and Vision Science, National Clinical Research Center for Ocular Diseases, Wenzhou Medical University

Address: 270 Xueyuan West Road, Lucheng District, Wenzhou, Zhejiang, China, 325027

Shiqi Zhou, MMed

Affiliation: School of Ophthalmology and Optometry and Eye Hospital, State Key Laboratory of Ophthalmology, Optometry and Vision Science, National Clinical Research Center for Ocular Diseases, Wenzhou Medical University

Address: 270 Xueyuan West Road, Lucheng District, Wenzhou, Zhejiang China, 325027

**Co-investigators:**

Fan Lu, PhD, Professor, Clinician Scientist

Affiliation: School of Ophthalmology and Optometry and Eye Hospital, State Key Laboratory of Ophthalmology, Optometry and Vision Science, National Clinical Research Center for Ocular Diseases, Wenzhou Medical University

Address: 270 Xueyuan West Road, Lucheng District, Wenzhou, Zhejiang China, 325027

Lihua Yu, MMed, Ophthalmologist and Optometrist
Affiliation: School of Ophthalmology and Optometry and Eye Hospital, State Key Laboratory of Ophthalmology, Optometry and Vision Science, National Clinical Research Center for Ocular Diseases, Wenzhou Medical University

Address: 270 Xueyuan West Road, Lucheng District, Wenzhou, Zhejiang China, 325027

1. **Study site**

The study will be conducted at the Affiliated Eye Hospital of Wenzhou Medical University. The hospital is among the three leading eye hospitals in China and is recognized for its comprehensive eye care services, advanced ophthalmology and optometry technologies, and high-level scientific research. As a major tertiary eye hospital in China, it serves a large number of patients nationwide.

1. **Background**

Myopia has become a global public health concern [1]. The World Health Organization (WHO) estimates that by 2050, nearly half of the global population will be affected by myopia, posing significant challenges to healthcare systems worldwide [2]. Owing to rapid lifestyle changes and increasing academic pressure, the prevalence of myopia and high myopia in China has risen annually, with a trend towards earlier onset in younger children [3]. Studies have shown that early-onset myopia is more likely to progress to high myopia [4], which is strongly associated with serious ocular diseases, including macular degeneration and retinal detachment [2,5–8,9]. Therefore, early intervention to prevent myopia is crucial for improving individual well-being and alleviating the burden on national healthcare systems.

Current interventions for myopia prevention remain limited. One of the most widely recognized preventive strategies is increasing outdoor activity [10–12]. However, the growing reliance on electronic devices and increasing academic demands have substantially reduced the time that children and adolescents spend outdoors. Although atropine has been used as a pharmacological intervention for myopia prevention [13–15], further research is required to determine its optimal dosage and appropriate target population. Recently, various types of functional lenses have been developed for myopia management. These lenses incorporate different mechanisms for myopia control, such as inducing peripheral myopic defocus [16] or higher-order aberrations [17,18]. However, most studies have focused on children who are already myopic. Whether these technologies can prevent myopia in children who have not yet developed it warrants further investigation.

We developed an aberration-enhanced dual-surface (AEDS) lens by combining two techniques that have been shown to be effective in myopia control—peripheral myopic defocus and aberration enhancement—which were incorporated into the anterior and posterior surface design of the lens, respectively. We plan to conduct a randomized controlled trial involving emmetropic and mildly hyperopic children to evaluate its effectiveness in preventing myopia.

1. **Hypothesis**

Hypothesis 1: AEDS lenses can effectively prevent the onset of myopia in non-myopic children.

Hypothesis 2: The effectiveness of AEDS lenses in preventing myopia in emmetropic children will be greater than that in mildly hyperopic children.

1. **Study objectives**

Primary objective: To evaluate the effectiveness of AEDS spectacle lenses in preventing myopia in non-myopic children.

Secondary objective: To examine the influence of baseline characteristics and daily behaviours on the effectiveness of AEDS spectacle lenses.

1. **Material and methods**

**6.1 Study design**

This study will be a 2-year, single-centre, double-masked, randomized controlled trial commencing in June 2023. Participants will be randomly assigned to either the intervention group or the control group and further stratified as emmetropic (−0.50 D < cycloplegic spherical equivalent refraction [SER] < +0.50 D) or mildly hyperopic (+0.50 D ≤ cycloplegic SER ≤ +2.00 D). Participants in the intervention group will wear AEDS lenses in their daily lives, whereas those in the control group will wear single-vision (SV) lenses.

- 1. **Scheme**

**Figure 1. Study flow diagram**


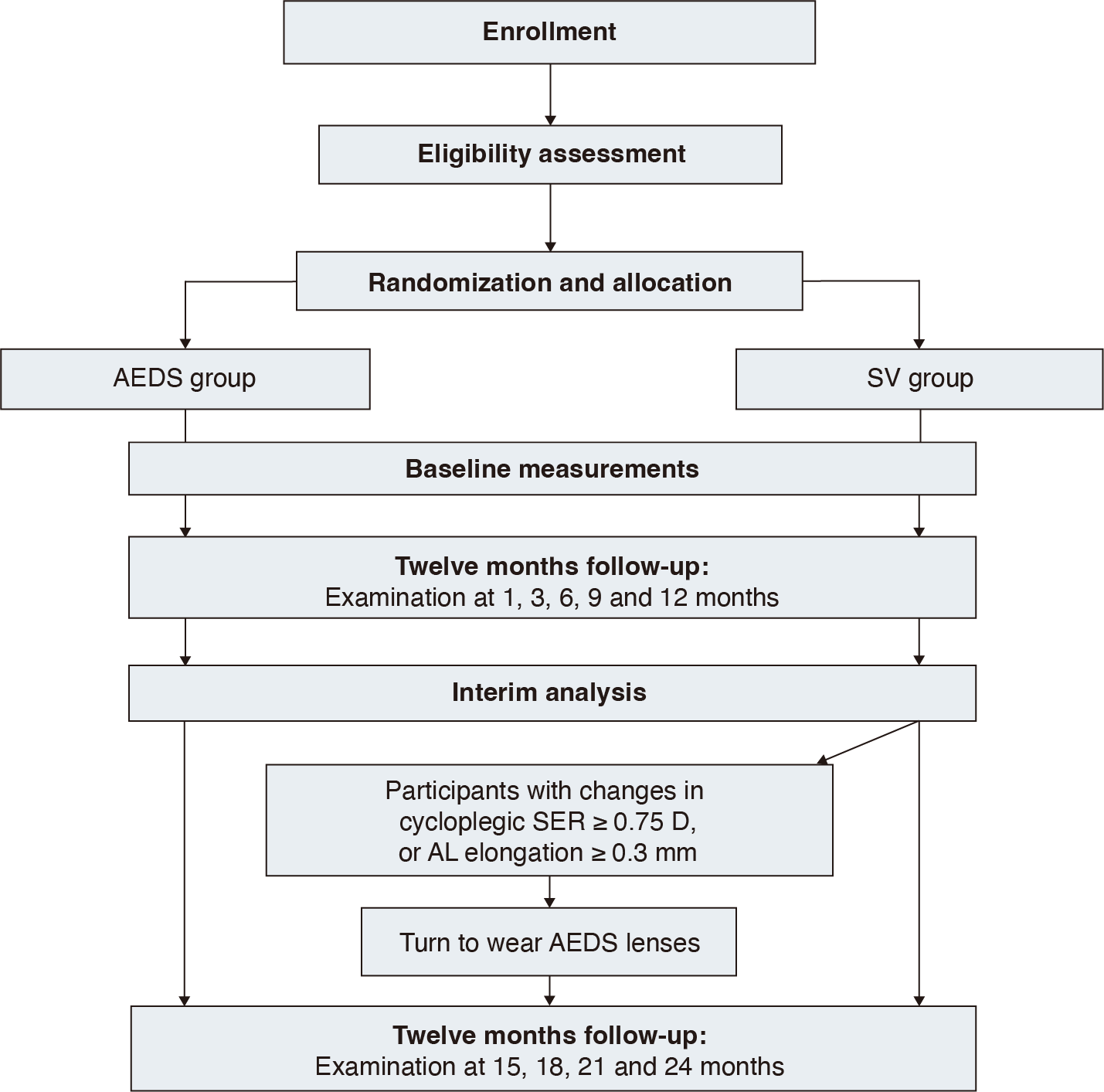


- 1. **Enrolment and eligibility**

Participants will be recruited from the hospital outpatient clinics. Advertisements will be displayed in the hospital’s public areas, and referrals from clinical optometrists will form part of the recruitment strategy.

Inclusion criteria: (1) age 6–12 years; (2) SER between −0.50 D and +2.00 D measured using an autorefractor under cycloplegia; (3) cycloplegic astigmatism ≤ 1.50 D; (4) distance best-corrected visual acuity (BCVA) of 0.1 logMAR or better; (5) anisometropia ≤ 1.50 D; (6) willingness to wear spectacle lenses.

Exclusion criteria: (1) intraocular pressure ≥ 21 mmHg; (2) manifest strabismus; (3) ocular or systemic diseases affecting visual development (excluding refractive errors); (4) history of ocular or systemic surgery affecting visual development; (5) use of defocus spectacle lenses or low-concentration atropine eye drops within the past 3 months; (6) use of orthokeratology lenses or other products intended to prevent or control myopia within the past 6 months (e.g., red light–based treatment methods); (7) history of systemic medications affecting growth and development (e.g., growth hormone); (8) concurrent participation in any clinical trial related to myopia control or prevention; (9) any other condition deemed unsuitable for participation by the investigator.

- 1. **Sample size**

The sample size was calculated using a two-sample t-test assuming equal variances in SPSS (IBM Corp., Armonk, NY, USA). The mean (standard deviation) annual axial elongation was assumed to be 0.36 (0.23) mm in the control group, based on previous studies of non-myopic children aged 6–12 years [13–15,19]. The intervention effect of the AEDS lens was assumed to be 40%. Allowing for a dropout rate of 10%, a minimum sample size of 94 participants (47 per group) was required to achieve 80% power at a two-sided significance level of 0.05.

- 1. **Randomisation and masking**

Randomisation will be conducted by a clinical research coordinator. Eligible participants will be randomly assigned in a 1:1 ratio to either the intervention group or the control group according to a computer-generated allocation sequence. The coordinator will notify the manufacturer of the appropriate lens type for each participant and will inspect the lenses upon receipt. All spectacle lenses will be packaged in identical eyeglass cases and provided by the coordinator to the dispensers, who will distribute them to the participants. The clinical research coordinator will not be involved in recruitment, clinical examinations, data collection, or data analysis. All other investigators (including examiners, dispensers, and statisticians), as well as participants and their parents or guardians, will remain masked throughout the study. To objectively assess whether blinding was inadvertently compromised, a formal evaluation will be conducted at the end of the study. Participants (or their guardians) and the primary outcome assessors will be asked to guess the assigned group. Statistical analysis will then be performed to determine whether the proportion of correct guesses differs significantly from that expected by chance (50%). The results of this blinding assessment will be presented in the final research report.

- 1. **Interventions**

Participants in the intervention group will wear AEDS spectacle lenses, whereas participants in the control group will wear SV spectacle lenses. The AEDS lens (Shanghai Baoli Lai Optical Lens Co. and Suzhou Mingshi Optical Technology Co., Ltd.) was designed by combining two technologies: a concentric microlens array on the anterior surface to generate peripheral myopic defocus, and a radial refractive gradient on the posterior surface to increase total ocular aberrations. The design is protected under the Patent Cooperation Treaty (202122046858X and 202221884655.6). Both the anterior and posterior surfaces of the lens incorporate a central optical zone (10.13 mm in diameter) designed to correct refractive error and provide a clear retinal image at the foveal centre. The peripheral zone of the anterior surface contains 11 concentric circular microlens arrays with different refractive powers, and the radius of the outermost ring is 59.57 mm. The refractive power of the lenslets is +5.00 D in the three inner rings, +5.50 D in the intermediate four rings, and +6.00 D in the four outer rings. The peripheral zone of the posterior surface incorporates a radial refractive gradient designed to enhance higher-order aberrations (e.g., spherical aberration and secondary astigmatism).

The following equation describes the Zernike polynomial representation of aberration-enhanced modulation on the posterior surface of the AEDS lens:

$$z=\frac{cr^{2}}{1+\sqrt{1-{(1+k)c}^{2}r^{2}}}+\sum_{i=1}^{N} 2\pi A_{i}Z_{i}(\rho,\varphi)$$

where z denotes the sagittal height of the surface, c is the vertex curvature, r represents the radial distance from the optical axis, k denotes the conic constant of the surface, N is the number of Zernike coefficients included in the series, i is the index of the Zernike polynomial term, ρ is the normalised radial coordinate, and φ is the angular coordinate. The normalised orthogonal polynomials expressed in polar coordinates are presented in Table 1, where x = ρcosφ, y = ρsinφ, ρ^2^ = x^2^ + y^2^, and n denotes the order of the polynomial.

**Table 1. Normalised orthogonal polynomials in polar coordinates**

| n | i | $Z_{i}(\rho,\varphi)$ |
| --- | --- | --- |
| 0 | 1 | 1 |
| 1 | 2 | $2\rho\cos\varphi$ |
| 1 | 3 | $2\rho\sin\varphi$ |
| 2 | 4 | $\sqrt{3}(2\rho^{2}-1)$ |
| 2 | 5 | $\sqrt{6}\rho^{2}\sin2\varphi$ |
| 2 | 6 | $\sqrt{6}\rho^{2}\cos2\varphi$ |
| 3 | 7 | $\sqrt{8}(3\rho^{3}-2\rho)\sin\varphi$ |
| 3 | 8 | $\sqrt{8}(3\rho^{3}-2\rho)\cos\varphi$ |
| 3 | 9 | $\sqrt{8}\rho^{3}\sin3\varphi$ |
| 3 | 10 | $\sqrt{8}\rho^{3}\cos3\varphi$ |
| 4 | 11 | $\sqrt{5}(6\rho^{4}-{6\rho}^{2}+1)$ |
| 4 | 12 | $\sqrt{10}(4\rho^{4}-{3\rho}^{2})\cos2\varphi$ |
| 4 | 13 | $\sqrt{10}(4\rho^{4}-{3\rho}^{2})\sin2\varphi$ |
| 4 | 14 | $\sqrt{10}\rho^{4}\cos4\varphi$ |
| 4 | 15 | $\sqrt{10}4\rho^{4}\sin4\varphi$ |
| ...... | ...... | ...... |

The following equation represents the Zernike polynomial corresponding to a selected configuration of the posterior surface. The magnitudes of the different aberrations at 10° and 15° field-of-view angles are presented in Table 2.

$$z= \frac{{cr}^{2}}{1+\sqrt{1-(1+k)c^{2}r^{2}}}+2\pi(2.3993Z_{1}-1.0182Z_{2}-0.0605Z_{3}+2.4179Z_{4}-0.2798Z_{5}-0.0062Z_{6}-0.2999Z_{7}-0.0170Z_{8}+0.0268Z_{9}+0.1145Z_{10}+0.0245Z_{11}-0.0332Z_{12}-0.0098Z_{13}+0.0626Z_{14}+0.0027Z_{15})$$

**Table 2. Magnitudes of different aberrations at 10° and 15° field-of-view angles**

| Aberration order | Zi | Zi at 10° Field-of-View Angle | Zi at 15° Field-of-View Angle | Aberration Description |
| --- | --- | --- | --- | --- |
| Second order | Z4 | −17.50865552 | −29.70500871 | Defocus |
|  | Z5 | −0.15539903 | −9.36105801 | Coma-like distortion |
|  | Z6 | −3.3065078 | −0.78442698 | Horizontal astigmatism |
| Third order | Z7 | −0.0710118 | −0.13242021 | Vertical coma |
|  | Z8 | −0.03149834 | −0.5278612 | Horizontal coma |
|  | Z9 | 0.01244168 | 0.26057668 | Oblique trefoil |
|  | Z10 | 0.03578069 | 0.14241967 | Vertical trefoil |
| Fourth order | Z11 | 0.12700155 | 0.15656005 | Spherical aberration |
|  | Z12 | 0.00157475 | 0.00321444 | Horizontal secondary astigmatism |
|  | Z13 | −0.0057844 | −0.01976174 | Oblique secondary astigmatism |
|  | Z14 | −0.00110142 | −0.00383473 | Horizontal quadrafoil |
|  | Z15 | 0.00092039 | 0.09126318 | Oblique quadrafoil |
| Fifth order | Z16 | −0.00012704 | 0.08969021 | Horizontal secondary coma |
|  | Z17 | −0.00325462 | 0.10228484 | Vertical secondary coma |
| … | … | … | … | … |

- 1. **Study outcomes**

Primary outcomes: changes in axial length (AL) and cycloplegic SER from baseline.

Secondary outcomes: incidence of myopia (defined as cycloplegic SER ≤ −0.50 D) and average daily lens-wearing time.

Safety outcomes: corneal astigmatism, total astigmatism, distance visual acuity, and participants’ subjective experiences while wearing the lenses.

**6.8 Study visit schedule**

Participants will undergo 24 months of follow-up during the trial. Table 3 presents the follow-up schedule and the examinations to be performed at each visit.

**Table 3. Examinations at baseline and at each follow-up visit**

| **Items** | **Baseline** | **1 month** | **3 months** | **6 months** | **9 months** | **12 months** | **15 months** | **18 months** | **21 months** | **24 months** |
| --- | --- | --- | --- | --- | --- | --- | --- | --- | --- | --- |
| Inclusion/exclusion criteria assessment | √ |  |  |  |  |  |  |  |  |  |
| Informed consent | √ |  |  |  |  |  |  |  |  |  |
| Slit-lamp biomicroscopy | √ |  |  | √ |  | √ |  | √ |  | √ |
| Subjective and objective refraction without cycloplegia | √ |  | √ | √ | √ | √ | √ | √ | √ | √ |
| Subjective and objective refraction with cycloplegia | √ |  |  | √ |  | √ |  | √ |  | √ |
| Monocular pupillary distance | √ |  | √ | √ | √ | √ | √ | √ | √ | √ |
| Spectacle lens dispensing | √ |  |  |  |  |  |  |  |  |  |
| Frame adjustment and pupil height measurement | √ | √ | √ | √ | √ | √ | √ | √ | √ | √ |
| Best-corrected visual acuity | √ |  | √ | √ | √ | √ | √ | √ | √ | √ |
| Intraocular pressure | √ |  |  |  |  | √ |  |  |  | √ |
| Strabismus at 33 cm | √ |  |  |  |  | √ |  |  |  | √ |
| Pupil size | √ |  |  |  |  | √ |  |  |  | √ |
| Axial length | √ | √ | √ | √ | √ | √ | √ | √ | √ | √ |
| Accommodative amplitude | √ |  |  |  |  | √ |  |  |  | √ |
| Accommodative facility | √ |  |  |  |  | √ |  |  |  | √ |
| Contrast sensitivity | √ |  |  | √ |  | √ |  |  |  | √ |
| Daily lens-wearing time |  | √ | √ | √ | √ | √ | √ | √ | √ | √ |
| Questionnaire on daily schedule |  | √ | √ | √ | √ | √ | √ | √ | √ | √ |
| Questionnaire on lens adaptation | √ | √ | √ |  |  |  |  |  |  |  |

**6.9 Outcome measurements**

Distance visual acuity will be measured, with and without spectacles, using a standard logarithmic visual acuity chart at a distance of 5 m. BCVA will be assessed after correction of the subjective refractive error.

Objective and subjective SER, both with and without cycloplegia, will be measured by a masked examiner. One drop of 0.5% proxymetacaine hydrochloride and three drops of 1% cyclopentolate will be administered to induce cycloplegia, with a 5-minute interval between each instillation. Refractive error will be measured at least 30 minutes after the administration of the eye drops to ensure complete cycloplegia, confirmed by pupil dilation (> 6 mm), absence of the light reflex, and lack of an accommodative response. Objective refractive error will then be measured using an autorefractor (Topcon KR-800, Topcon Corporation, Tokyo, Japan), and the averaged value (including total astigmatism) will be calculated as the mean of ten consecutive measurements. Subjective SER will be measured using a phoropter (Topcon Corporation, Tokyo, Japan). Lenses will be replaced if the change in subjective SER exceeds 0.25 D or if there are scratches on the lens surface.

Mean AL and corneal astigmatism will be calculated as the average of five consecutive measurements obtained using an IOLMaster (Carl Zeiss Meditec, Jena, Germany).

Participants’ daily schedules and subjective experiences while wearing the lenses will be recorded using self-administered questionnaires (Table S1 in Additional file 2), which consist of items on lens-wearing duration (Items 1–2), daily schedule (Items 3–8), and adaptation to the lenses (Items 9–10). The average daily lens-wearing time over 12 months (T12) and 24 months (T24) will be calculated using the following formulae:

*T_12_ = (**a_1_ × 30 + a_2_× 60 + a_3_ × 90 + a_4_ × 90 + a_5_ × 90)/360*

*T_24_ =0.5× [T_12_+(a_6_ + a_7_ + a_8_ + a_9_)* *× 90/360]*

In these equations, *a_1_* to *a_9_* represent the average daily lens-wearing time reported at each follow-up visit (from 1 month to 24 months). *T_12_* represents the average daily lens-wearing time over the first 12 months, and *T_24_* represents the average daily lens-wearing time over 24 months.

The average daily lens-wearing time at each visit will be calculated using the following formula:

*Average daily lens-wearing time at each visit = (b × d_1_ + c × d_2_)/(d_1_+d_2_)*

In this equation, *b* denotes the average daily lens-wearing time on workdays, whereas *c* denotes the average daily lens-wearing time on weekends or holidays, both reported at each visit. *d_1_* and *d_2_* represent the total number of workdays and weekends (or holidays), respectively, between the current follow-up visit and the previous visit. The average daily hours spent on outdoor activities, electronic device use, and sleep will also be calculated using a similar formula.

1. **Data management**

The study will use paper-based documentation for data collection. Each participant will be assigned a unique screening identifier to facilitate accurate data retrieval and tracking. In compliance with national regulations, all study documents and publications will be encrypted or de-identified to safeguard participant confidentiality.

Data from each follow-up visit must be entered into the data collection system within seven days to ensure timely and accurate data management. After the 12-month follow-up visit, outcome data and group allocation will be provided to the data analysts for an interim analysis, following removal of identifiable information, to assess the preliminary efficacy and safety of the AEDS lens. Masking will be maintained for data collectors and participants. After completion of the 2-year follow-up, data managers will perform comprehensive logical checks, and all identified discrepancies will be resolved before database lock. Once the data collection system has been locked, the final version of the data management report will be generated. All data will be reviewed by the principal investigator, sponsor, statisticians, and monitors under masked conditions. The dataset will then be transferred to the statistical team for formal analysis in accordance with the pre-specified statistical analysis plan.

**8. Statistical analysis**

Statistical analysis will be performed using SPSS software (IBM Corp., Armonk, NY, USA). The distribution of continuous variables will be assessed using the Kolmogorov–Smirnov test. Intergroup and inter-subgroup differences in continuous variables will be analysed using independent-samples t-tests. Categorical variables will be analysed using the chi-square test. A univariate general linear model will be applied to evaluate changes in AL and SER, adjusting for sex, age, lens-wearing time, baseline AL (or baseline cycloplegic SER), photopic pupil size, daily duration of outdoor activities, duration of electronic device use, and the number of myopic parents. Both adjusted and unadjusted analyses will be conducted to ensure the robustness of the assessment of intergroup differences. Subgroup analyses will be conducted according to age, baseline refractive status (emmetropia and mild hyperopia), and daily lens-wearing time. The correlation between axial elongation and changes in refraction will be analysed using Pearson’s correlation analysis. To reduce the risk of Type I error resulting from multiple comparisons, a *P*-value < 0.025 will be considered statistically significant for analyses of the 6-month follow-up data and subgroup analyses. As the study is exploratory in nature, no adjustments for multiple testing in subgroup analyses or imputation for missing data were specified at the design stage. In all other analyses, a *P*-value < 0.05 will be considered statistically significant.

**9.** **Ethics and governance**

Written informed consent will be obtained from each participant and their parents or guardians after the objectives, procedures, potential risks, and benefits of the study have been explained. Signed copies of the consent form will be provided to participants and retained in the study records. All study documents, including this protocol, the consent forms, recruitment advertisements, and any amendments, will be reviewed and approved by the Ethics Committee of the Eye Hospital of Wenzhou Medical University and registered with the Chinese Clinical Trial Registry. The study is designed in accordance with the WHO definition of a clinical trial and will be conducted in accordance with the principles of the Declaration of Helsinki.

**References**

1. Sankaridurg P, Tahhan N, Kandel H, Naduvilath T, Zou H, Frick KD, et al. IMI impact of myopia. Invest Ophthalmol Vis Sci. 2021;62(5):2.

2. Holden BA, Fricke TR, Wilson DA, Jong M, Naidoo KS, Sankaridurg P, et al. Global prevalence of myopia and high myopia and temporal trends from 2000 through 2050. Ophthalmology. 2016;123(5):1036-42.

3. Jonas JB, Ang M, Cho P, Guggenheim JA, He MG, Jong M, et al. IMI prevention of myopia and its progression. Invest Ophthalmol Vis Sci. 2021;62(5):6.

4. Lai H, Gao K, Li M, Li T, Zhou X, Zhou X, et al. Handling missing data and measurement error for early-onset myopia risk prediction models. BMC Med Res Methodol. 2024;24(1):194.

5. Mu J, Zeng D, Fan J, Liu M, Jiang M, Shuai X, et al. Epidemiological characteristics and influencing factors of myopia among primary school students in Southern China: a longitudinal study. Int J Public Health. 2023;68:1605424.

6. Liu W, Gong L, Li Y, Zhu X, Stewart JM, Wang C. Peripapillary atrophy in high myopia. Curr Eye Res. 2017;42(9):1308-12.

7. Lim DH, Lyu IJ, Choi SH, Chung ES, Chung TY. Risk factors associated with night vision disturbances after phakic intraocular lens implantation. Am J Ophthalmol. 2014;157(1):135-41.e1.

8. Pärssinen O, Kauppinen M. Risk factors for high myopia: a 22-year follow-up study from childhood to adulthood. Acta Ophthalmol. 2019;97(5):510-8.

9. Oncel D, Minaker S, Shepherd EA, Rezaei S, Boucher N, Aggarwal N, et al. Risk factors for proliferative vitreoretinopathy in a large clinical database. Retina. 2025;45(5):818-26.

10. Rose KA, Morgan IG, Ip J, Kifley A, Huynh S, Smith W, et al. Outdoor activity reduces the prevalence of myopia in children. Ophthalmology. 2008;115(8):1279-85.

11. Galvis V, Tello A, Gómez LM, Camacho PA, Ortiz RG. Re: Wu et al.: Myopia prevention and outdoor light intensity in a school-based cluster randomized trial (Ophthalmology. 2018;125:1239–1250). Ophthalmology. 2018;125(11):e77.

12. Xiong S, Sankaridurg P, Naduvilath T, Zang J, Zou H, Zhu J, et al. Time spent in outdoor activities in relation to myopia prevention and control: a meta-analysis and systematic review. Acta Ophthalmol. 2017;95(6):551-66.

13. Yam JC, Zhang XJ, Zhang Y, Yip BHK, Tang F, Wong ES, et al. Effect of low-concentration atropine eyedrops vs. placebo on myopia incidence in children: the LAMP2 randomized clinical trial. JAMA. 2023;329(6):472-81.

14. Jethani J. Efficacy of low-concentration atropine (0.01%) eye drops for prevention of axial myopic progression in premyopes. Indian J Ophthalmol. 2022;70(1):238-40.

15. Wang W, Zhang F, Yu S, Ma N, Huang C, Wang M, et al. Prevention of myopia shift and myopia onset using 0.01% atropine in premyopic children - a prospective, randomized, double-masked, and crossover trial. Eur J Pediatr. 2023;182(6):2597-606.

16. Su B, Cho P, Vincent SJ, Zheng J, Chen J, Ye C, et al. Novel Lenslet-ARray-Integrated spectacle lenses for myopia control: a 1-year randomized, double-masked, controlled trial. Ophthalmology. 2024;131(12):1389-97.

17. Liu X, Wang P, Xie Z, Sun M, Chen M, Wang J, et al. One-year myopia control efficacy of cylindrical annular refractive element spectacle lenses. Acta Ophthalmol. 2023;101(6):651-7.

18. Lau JK, Vincent SJ, Collins MJ, Cheung SW, Cho P. Ocular higher-order aberrations and axial eye growth in young Hong Kong children. Sci Rep. 2018;8(1):6726.

19. Li SM, Ran AR, Kang MT, Yang X, Ren MY, Wei SF, et al. Effect of text messaging parents of school-aged children on outdoor time to control myopia: a randomized clinical trial. JAMA Pediatr. 2022;176(11):1077-83.
