# Supplementary material for: Dual-surface aberration-increasing lenses versus single-vision lenses in non-myopic children: a randomized clinical trial
Source: Eye Vis (Lond). 2026 Apr 27;13:15. doi: 10.1186/s40662-026-00486-0 (PMC13112723; doi:10.1186/s40662-026-00486-0)
Supplement: Supplementary file 2 — Supplementary Material 2. [file 40662_2026_486_MOESM2_ESM.docx]

**Supplementary online content**

- **Table S1** Self-assessment questionnaires
- **Table S2** Eye habits of participants during the 12-month follow-up
- **Table S3** Unadjusted axial elongation and refractive change in each group at the 6-month follow-up
- **Table S4** Results at the 12-month follow-up of subgroup analyses based on age and daily lens-wearing time
- **Table S5** Subjective adaptation and visual quality at baseline and at the 1-month follow-up
- **Table S6** Mean change of area under the log contrast sensitivity function (AULCSF) in mesopic and photopic conditions
- **Figure S1** Illustration of the dual-surface aberration-increasing lens
- **Figure S2** Sagittal height map of the posterior surface
- **Figure S3** Modulation transfer function of the dual-surface aberration- increasing lens and the single-surface lens with microlens arrays.
- **Figure S4** Distribution of baseline cycloplegic spherical equivalent refraction (SER) in the DSAI (**a**) and the SV group (**b**).
- **Figure S5** Proportion of participants with different degrees of axial elongation (**a**) and refractive change (**b**) at the 12-month follow-up.
- **Formulae for calculating the average daily hours spent wearing lenses, sleeping, using electronic devices, and engaging in outdoor activities**
- **Factors associated with model-adjusted axial elongation and refractive change in emmetropic children**

**Table S1.** Self-assessment questionnaires

| **Questionnaire on lens-wearing time**   1. How many hours do you wear your spectacle lenses on each workday (Monday to Friday)? 2. How many hours do you wear your spectacle lenses on each weekend day (Saturday and Sunday) or on holidays?   (Please indicate any special circumstances, e.g., if the spectacles were broken or forgotten.) |
| --- |
| **Questionnaire on daily schedules**   1. How much time do you spend outdoors on each workday (Monday to Friday)? 2. At what time do you go to bed and wake up on each workday (Monday to Friday)? 3. How much time do you spend using electronic devices on each workday (Monday to Friday)? 4. How much time do you spend outdoors on each weekend day (Saturday and Sunday) or on holidays? 5. At what time do you go to bed and wake up on each weekend day (Saturday and Sunday) or on holidays?   8. How much time do you spend using electronic devices on each weekend day (Saturday and Sunday) or on holidays? |
| **Questionnaire on lens adaptation**   1. Do you experience discomfort while wearing your spectacle lenses (e.g., nausea, dizziness, headache, or eye strain)?   0 Very uncomfortable  1 Slightly uncomfortable  2 Mildly uncomfortable  3 No discomfort   1. Do you see clearly at both distance and near while wearing your spectacle lenses?   0 very unclear 1 slightly clear 2 moderately unclear 3 very clear |

**Table S2.** Eye habits of participants during the 12-month follow-up

| **Items** | **DSAI group**  **(n = 46)** | **SV group**  **(n = 46)** | **df** | **t value** | ***P* value** |
| --- | --- | --- | --- | --- | --- |
| Average time spent on outdoor activities (hours/day) | 1.57 [0.45] | 1.72 [0.50] | 90 | 1.55 | 0.12 |
| Average sleep duration (hours/day) | 9.64 [0.84] | 9.43 [0.51] | 90 | −1.46 | 0.15 |
| Average time spent using electronic devices (hours/day) | 0.44 [0.32] | 0.55 [0.40] | 90 | 1.47 | 0.14 |
| Average lens-wearing time (hours/day) | 11.09 [1.07] | 10.97 [1.46] | 90 | −0.43 | 0.67 |
| *DSAI* = dual-surface aberration-increasing; *SV* = single-vision; *df* = degrees of freedom. Data are presented as mean [standard deviation (SD)]. | | | | | |

**Table S3.** Unadjusted axial elongation and refractive change in each group at the 6-month follow-up

| **Outcome measures** | **DSAI group**  **(n = 46)** | **SV group**  **(n = 46)** | **Mean difference** | **95% CI** | **df** | **t value** | ***P* value** |
| --- | --- | --- | --- | --- | --- | --- | --- |
| Axial elongation (mm) | 0.15 ± 0.02 | 0.24 ± 0.02 | 0.09 ± 0.03 | 0.04 to 0.15 | 90 | 3.55 | **0.001** |
| Refractive change (D) | −0.17 ± 0.05 | −0.41 ± 0.07 | −0.24 ± 0.08 | −0.40 to −0.07 | 90 | −2.85 | **0.005** |

*DSAI* = dual-surface aberration-increasing; *SV* = single-vision; *CI* = confidence interval; *df* = degrees of freedom; *D* = diopter; Data are presented as mean ± standard error (SE). *P* values in bold indicate statistical significance.

**Table S4.** Results at the 12-month follow-up of subgroup analyses based on age and daily lens-wearing time

| **Subgroup** | **DSAI group** | **SV**  **group** | | **Mean difference** | **95% CI** | **df** | **F value** | ***P value*** |
| --- | --- | --- | --- | --- | --- | --- | --- | --- |
| **Axial elongation (mm)** | | | | | | | | |
| Age (years) | | | | | | | | |
| 6.0–8.0 | 0.31 ± 0.05  (n = 23) | 0.39 ± 0.07  (n = 20) | 0.08 ± 0.08 | | −0.07 to 0.23 | 1 | 1.10 | 0.30 |
| 8.1–12.0 | 0.18 ± 0.04  (n = 23) | 0.37 ± 0.04  (n = 26) | 0.19 ± 0.05 | | 0.09 to 0.30 | 1 | 13.47 | **< 0.001** |
| Lens-wearing time (hours/day) | | | | | | | | |
| < 11 | 0.25 ± 0.05  (n = 20) | 0.37 ± 0.06  (n = 17) | 0.12 ± 0.07 | | −0.03 to 0.28 | 1 | 2.75 | 0.10 |
| ≥ 11 | 0.22 ± 0.04  (n = 26) | 0.37 ± 0.05  (n = 29) | 0.15 ± 0.06 | | 0.04 to 0.26 | 1 | 7.41 | **0.009** |
| **Refractive change (D)** | | | | | | | | |
| Age (years) | | | | | | | | |
| 6.0–8.0 | −0.36 ± 0.17  (n = 23) | −0.44 ± 0.20 (n = 20) | −0.08 ± 0.22 | | −0.54 to 0.38 | 1 | 0.13 | 0.73 |
| 8.1–12.0 | −0.24 ± 0.10  (n = 23) | −0.62 ± 0.11 (n = 26) | −0.38 ± 0.14 | | −0.65 to −0.10 | 1 | 7.62 | **0.009** |
| Lens-wearing time (hours/day) | | | | | | | | |
| < 11 | −0.41 ± 0.12  (n = 20) | −0.57 ± 0.13 (n = 17) | −0.16 ± 0.17 | | −0.52 to 0.20 | 1 | 0.86 | 0.36 |
| ≥ 11 | −0.22 ± 0.14  (n = 26) | −0.47 ± 0.15 (n = 29) | −0.26 ± 0.17 | | −0.60 to 0.08 | 1 | 2.42 | 0.13 |

*DSAI* = dual-surface aberration-increasing; *SV* = single-vision; *CI* = confidence interval; *df* = degrees of freedom; *D* = diopter. Data are presented as mean ± standard error (SE), adjusted for confounders including sex, age, lens-wearing time, baseline axial length (or baseline cycloplegic spherical equivalent refraction), photopic pupil size, number of myopic parents, and daily duration of outdoor activities and electronic device use. *P* values in bold indicate statistical significance.

**Table S5.** Subjective adaptation and visual quality at baseline and at the 1-month follow-up

| **Assessment items** | **DSAI group** | **SV group** | **X^2^** | ***P* value** |
| --- | --- | --- | --- | --- |
| Subjective comfort while wearing lenses at dispensing | | | | |
| Slightly uncomfortable | 3 (6%) | 3 (6%) | 0.00 | > 0.99 |
| No discomfort | 47 (94%) | 47 (94%) |  |  |
| Subjective comfort while wearing lenses at the 1-month visit | | | | |
| Slightly uncomfortable | 2 (4%) | 0 (0%) | 0.49 | 0.48 |
| No discomfort | 46 (96%) | 47 (100%) |  |  |
| Clarity of distance and near vision with lenses at the 1-month visit | | | | |
| Very clear | 48 (100%) | 47 (100%) | 0.00 | > 0.99 |

*DSAI* = dual-surface aberration-increasing; *SV* = single-vision. Data are presented as number (%).

**Table S6.** Mean change of area under the log contrast sensitivity function (AULCSF) in mesopic and photopic conditions

| **Binocular AULCSF** | **DSAI group** | **SV group** | **df** | **t value** | ***P* value** |
| --- | --- | --- | --- | --- | --- |
| **Mesopic (3 cd/m²)** | | | | | |
| 6-month follow-up | 0.03 [0.16] | 0.05 [0.18] | 90 | 0.70 | 0.50 |
| 12-month follow-up | 0.01 [0.15] | 0.02 [0.11] | 90 | 0.50 | 0.62 |
| **Photopic (85** **cd/m²)** | | | | | |
| 6-month follow-up | 0.05 [0.13] | 0.07 [0.21] | 90 | 0.44 | 0.66 |
| 12-month follow-up | 0.04 [0.13] | 0.04 [0.13] | 90 | -0.13 | 0.89 |

*DSAI* = dual-surface aberration-increasing; *SV* = single-vision; *df* = degrees of freedom; *cd/m^2^* = candela per square meter. Contrast sensitivity was measured at spatial frequencies of 3, 6, 12, and 18 cycles per degree at a viewing distance of 2.5 m. Data are presented as mean [standard deviation (SD)].

**Figure S1.** Illustration of the dual-surface aberration-increasing lens


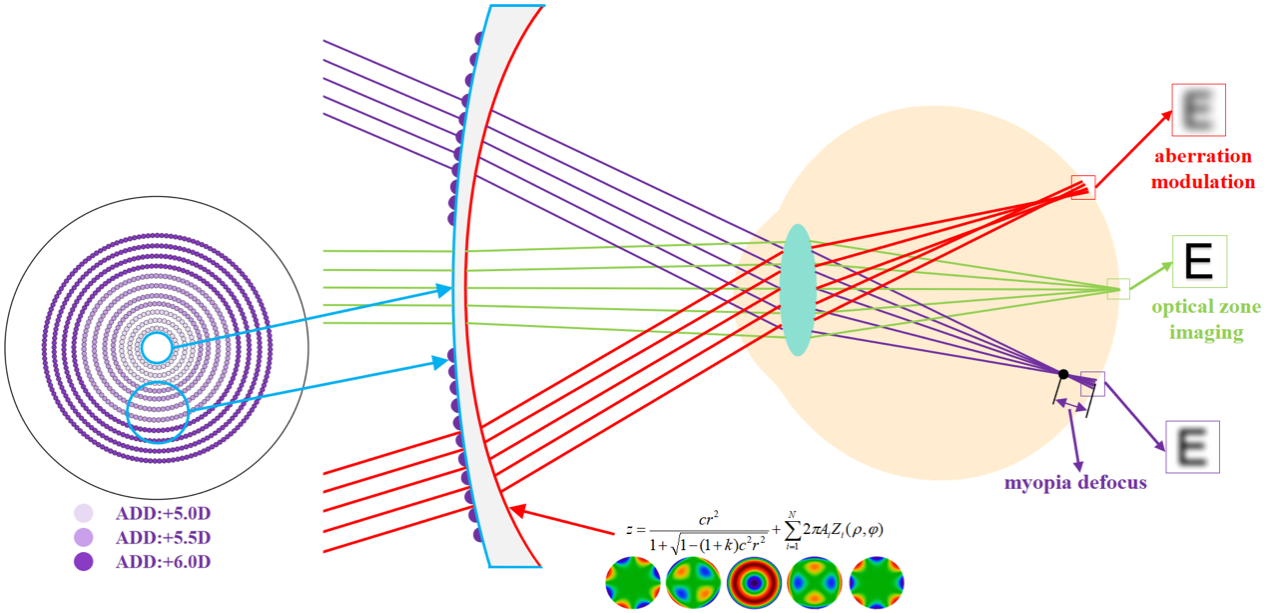


The anterior surface of the lens is shown on the left side of the figure. The eleven purple rings represent the concentric microlens array, and the different shades of purple indicate varying refractive powers of the lenslets. The radius of the outermost ring is 59.57 mm. The green lines represent light transmitted through the central optical zone (10.13 mm), forming a clear image on the retina. The purple lines illustrate the optical path when light is transmitted solely through the anterior surface of the lens, generating myopic defocus signals in front of the peripheral retina. In the side-profile illustration, the posterior surface is depicted in red. It features a radial refractive gradient in the peripheral region, which increases ocular aberrations. The formula represents the Zernike polynomial describing aberration modulation of the posterior surface, where *z* denotes the sagittal height, *c* is the vertex curvature, *r* represents the radial distance from the optical axis, *k* is the conic constant of the surface, *N* is the number of Zernike coefficients in the series, *i* is the index of the Zernike polynomial term, *ρ* is the normalised radial coordinate, and *φ* is the angular coordinate. Below the formula, examples of aberrations introduced by the posterior surface modulation are presented (e.g., spherical aberration and secondary astigmatism at 0° and 45°).

The Zernike polynomial corresponding to a selected instance of the posterior surface is presented below.

$$z= \frac{{cr}^{2}}{1+\sqrt{1-(1+k)c^{2}r^{2}}}+2\pi(2.3993Z_{1}-1.0182Z_{2}-0.0605Z_{3}+2.4179Z_{4}-0.2798Z_{5}-0.0062Z_{6}-0.2999Z_{7}-0.0170Z_{8}+0.0268Z_{9}+0.1145Z_{10}+0.0245Z_{11}-0.0332Z_{12}-0.0098Z_{13}+0.0626Z_{14}+0.0027Z_{15})$$

**Figure S2.** Sagittal height map of the posterior surface. **a** illustrates the three-dimensional sagittal height map of the posterior surface. Diopter refers to the refractive power of the posterior surface. **b** illustrates the two-dimensional sagittal height projection of the posterior surface when viewed perpendicularly.


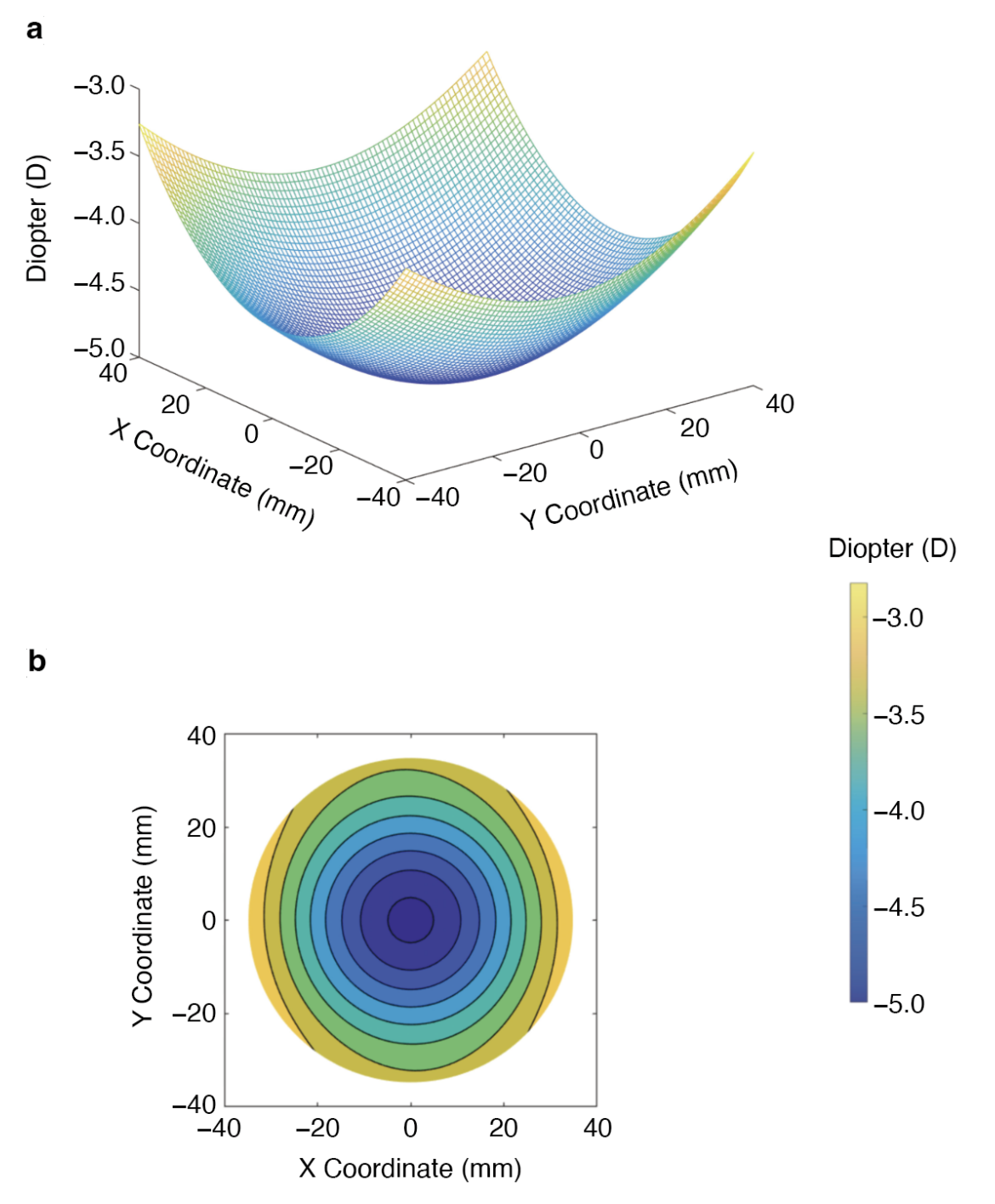


**Figure S3.** Modulation transfer function of the dual-surface aberration- increasing lens and the single-surface lens with microlens arrays. The modulation transfer function (MTF) was measured for a narrow beam along the tangential and sagittal meridians. The curves were calculated using the optical design software ZEMAX.


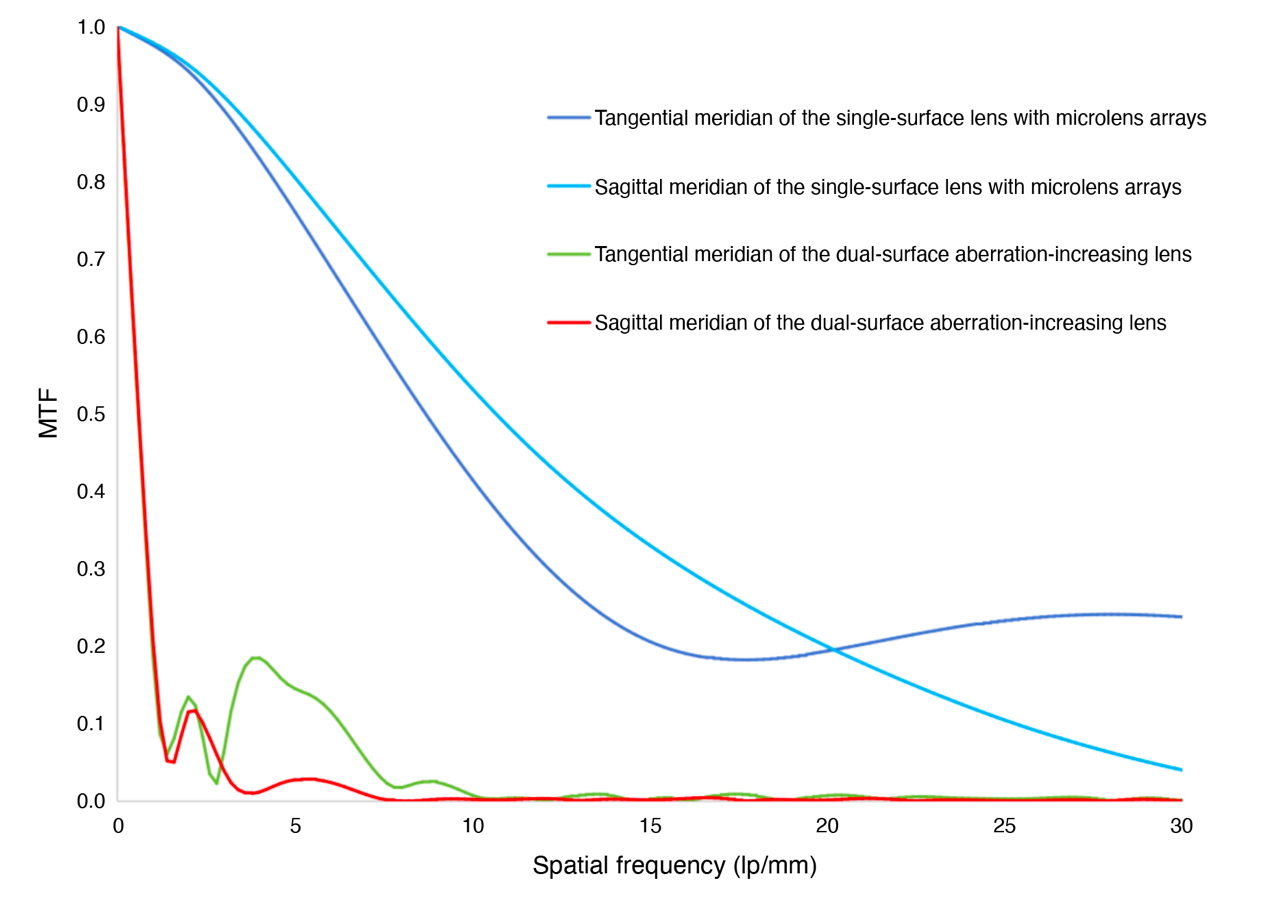
**Figure S4.** Distribution of baseline cycloplegic spherical equivalent refraction (SER) in the DSAI (**a**) and the SV group (**b**). DSAI, dual-surface aberration-increasing; SV, single-vision; SER, spherical equivalent refraction; D, diopter.

**
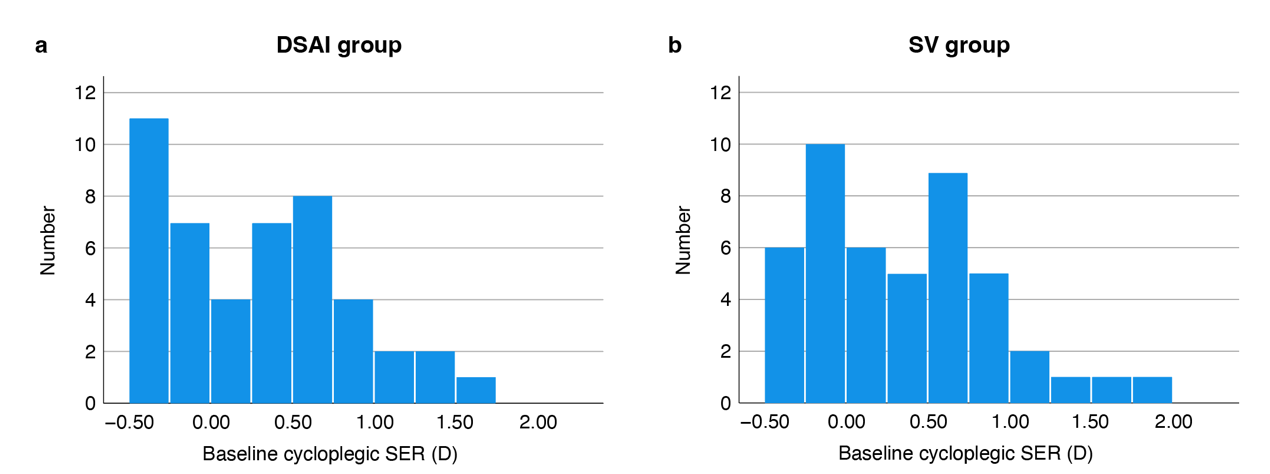
**

**Figure S5.** Proportion of participants with different degrees of axial elongation (**a**) and refractive change (**b**) at the 12-month follow-up. DSAI, dual-surface aberration-increasing; SV, single-vision; D, diopter. * *P* < 0.05.


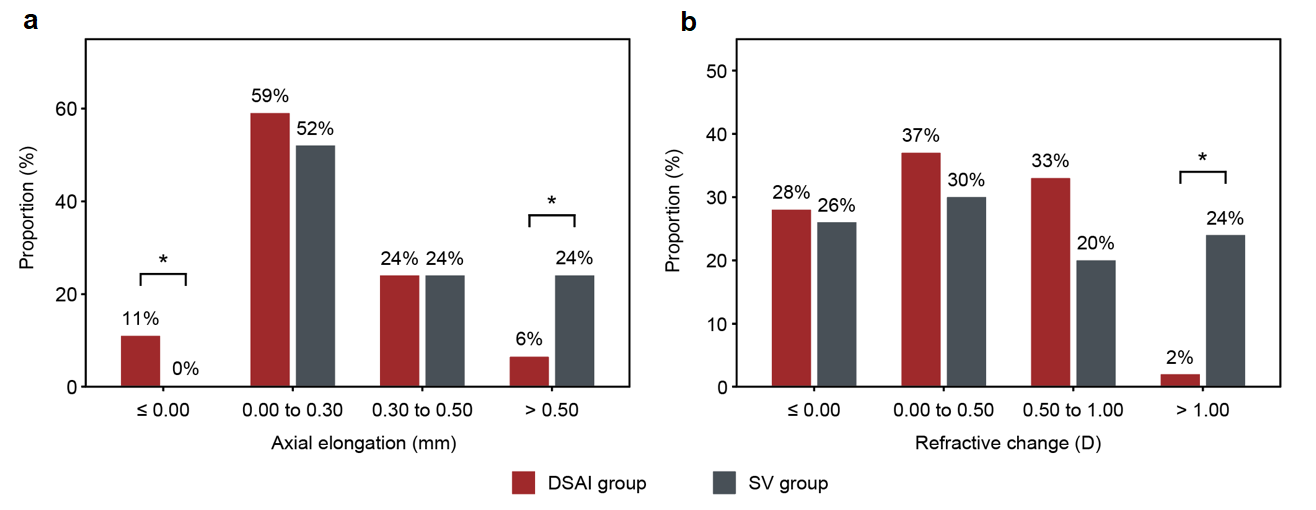
**Formulae for calculating the average daily hours spent wearing lenses, sleeping, using electronic devices, and engaging in outdoor activities**

The average daily lens-wearing time at each follow-up visit was calculated using the following formula:

*Average daily lens-wearing time reported at each visit = (a × c_1_ + b× c_2_)/(c_1_+c_2_)*

*a* represents the average daily lens-wearing time on workdays, whereas *b* represents the average daily lens-wearing time on weekends (or holidays), both reported at each follow-up visit. *c_1_* and *c_2_* denote the total number of workdays and weekend days (or holidays) between the current and previous follow-up visits, respectively.

The formulae for calculating the average daily lens-wearing time over 12 months and 24 months are as follows:

*T_12_ = (d_1_ × 30 + d_2_× 60 + d_3_ × 90 + d_4_ × 90 + d_5_ × 90)/360*

*T_24_ =0.5× [T_12_+(d_6_ + d_7_ + d_8_ + d_9_) × 90/360]*

*d_1_* to *d_9_* represent the average daily lens-wearing time reported at each follow-up visit (from 1-month to 24-month visits). *T_12_* and *T_24_* represent the average daily lens-wearing time over 12 and 24 months, respectively.

The average daily hours spent on outdoor activities, electronic device use, and sleep were also calculated using formulae analogous to those described above.

**Factors associated with model-adjusted axial elongation and refractive change in emmetropic children**

In the univariate general linear model analysis, group (*P* < 0.001), sex (*P* = 0.04), age (*P* = 0.01), and pupil size (*P* = 0.005) were significantly associated with axial elongation. Group (*P* = 0.01), sex (*P* = 0.04), and pupil size (*P* = 0.002) were significantly associated with refractive change.

Given the potential influence of pupil size on the effect of myopia control lenses [1], a multiple linear regression model was employed to clarify the direction of the associations between pupil size and the main outcome measures.

Larger pupil size was associated with slower myopic progression in the SV group (axial elongation: B = −0.12, *P* = 0.01; refractive change: B = 0.49, *P* < 0.001), whereas this pattern was not observed in the DSAI group (axial elongation: B = 0.05, *P* = 0.93; refractive change: B = −0.08, *P* = 0.63). In the present study, pupil size was not significantly associated with the efficacy of DSAI lenses in emmetropic children. Further studies with larger sample sizes are warranted to confirm this relationship.

**References**

1. Zhu J, Yang Z, Sun D, He T, Lin J, Dong Y, et al. Association Between Pupil Area and the Efficacy of Peripheral Defocus Spectacle Lenses in Myopia Control. Trans Vis Sci Tech. 2026;15:9.
